# Redetermined structure of 4-(benz­yloxy)benzoic acid

**DOI:** 10.1107/S2414314624007521

**Published:** 2024-08-06

**Authors:** Qiuyi Song, Sihui Long

**Affiliations:** a School of Chemical Engineering and Pharmacy Wuhan Institute of Technology,Wuhan Hubei 430205 People’s Republic of China; b School of Chemical Engineering and Pharmacy Wuhan Institute of Technology,Wuhan Hubei 430205 China; University of Aberdeen, United Kingdom

**Keywords:** synthon, hydrogen bond, acid-acid dimer, crystal structure

## Abstract

In the title compound, C_14_H_14_O_3_, the dihedral angle between the aromatic rings is 39.76 (9)°. In the crystal, the mol­ecules associate to form centrosymmetric acid–acid dimers linked by pairwise O—H⋯O hydrogen bonds. The precision of the geometric parameters in the present single-crystal study is about an order of magnitude better than the previous powder diffraction study [Chattopadhyay *et al.* (2013 ▸). *CrystEngComm*, **15**, 1077–1085].

## Abstract 2

The mol­ecules of the title compound form acid–acid homodimers in the crystal structure.

## Graphical abstract

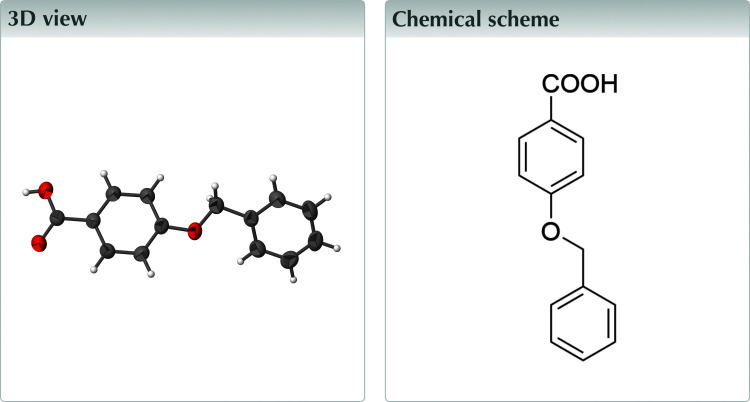

## Structure description

Non-steroidal anti-inflammatory drugs (NSAIDs) constitute approximately 5–10% of all prescribed medicines worldwide as anti­pyretic, anti-inflammatory and analgesic agents (Sohail *et al.*, 2023 ▸). Moreover, they are found to have a protective role against various critical diseases, such as cancer and cardiovascular diseases (Prasher & Sharma, 2021 ▸). It is estimated that 30 million individuals use NSAIDs daily (Bhala *et al.*, 2013 ▸).

**Figure 1:**
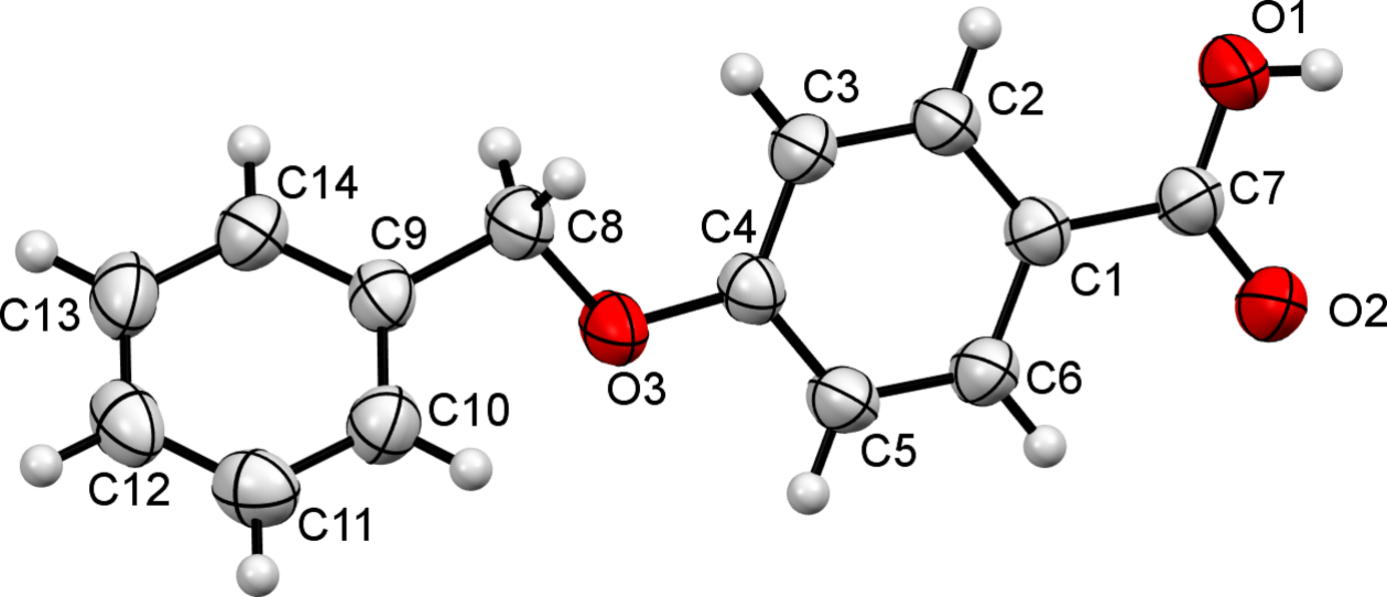
The mol­ecular structure of the title compound with displacement ellipsoids drawn at the 50% probability level.

As part of our studies in this area, the title compound (Fig. 1 ▸) was synthesized by a two-step reaction. The C1–C6 and C9–C14 aromatic rings subtend a dihedral angle of 39.76 (9)° and the linking C4—O3—C8—C9 bond has an *anti* conformation [torsion angle = −171.59 (12)°]. The short C4—O3 bond length of 1.3601 (16) Å indicates some conjugation of the O atom lone pair with the adjacent aromatic ring.

**Figure 2:**
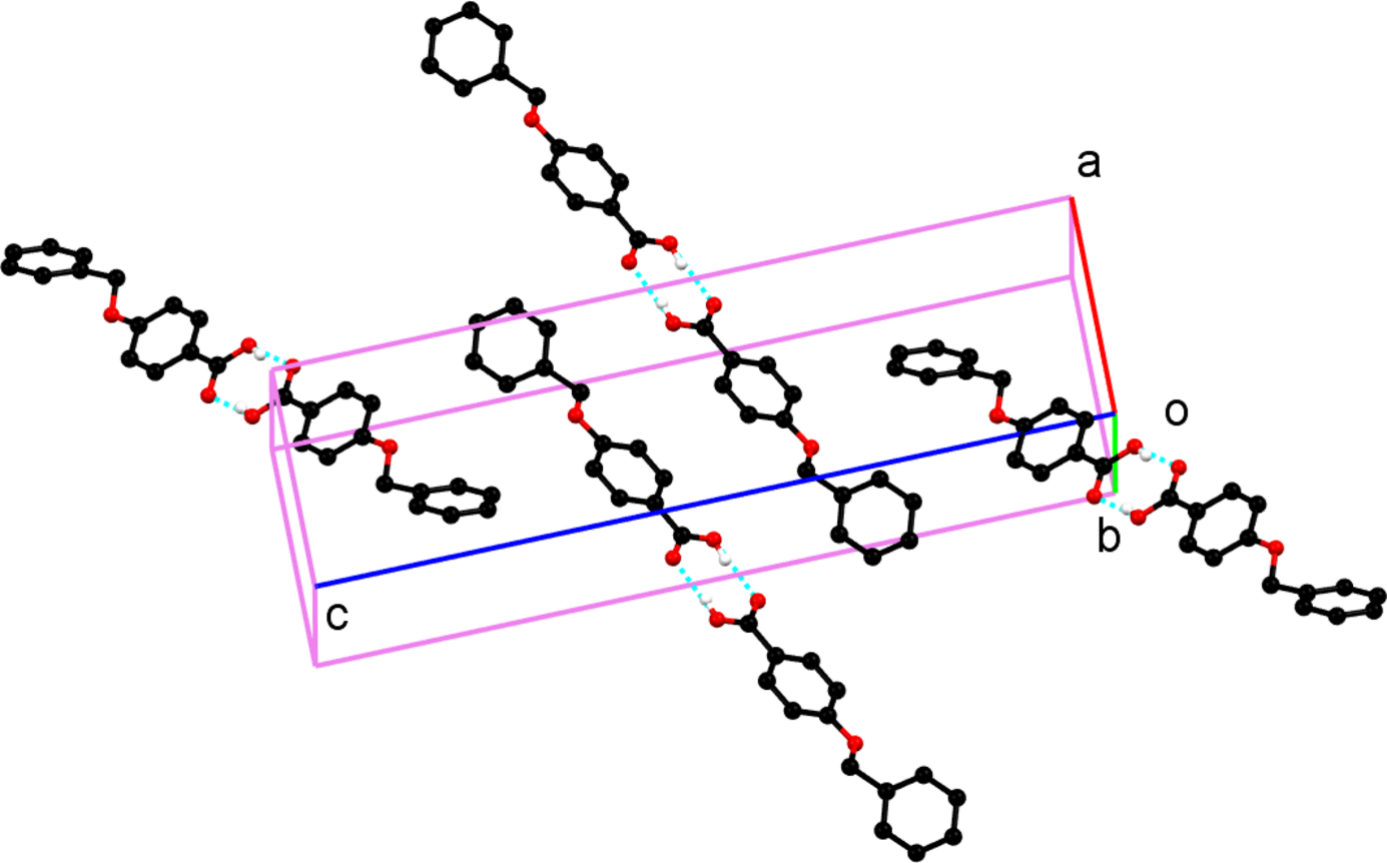
Packing of the mol­ecules in the title compound (for clarity, H atoms not involved in hydrogen bonding are omitted).

In the extended structure, mol­ecules pair up to form carb­oxy­lic acid–carb­oxy­lic acid hydrogen-bonded dimers (Table 1 ▸, Fig. 2 ▸). The crystal structure of the title compound was also solved from X-ray powder diffraction data (Chattopadhyay *et al.*, 2013 ▸) with corresponding hydrogen-bond parameters of 1.94 Å and 176°, respectively, which deviate from those obtained in this study from single-crystal X-ray diffraction.

## Synthesis and crystallization

**Figure 3:**
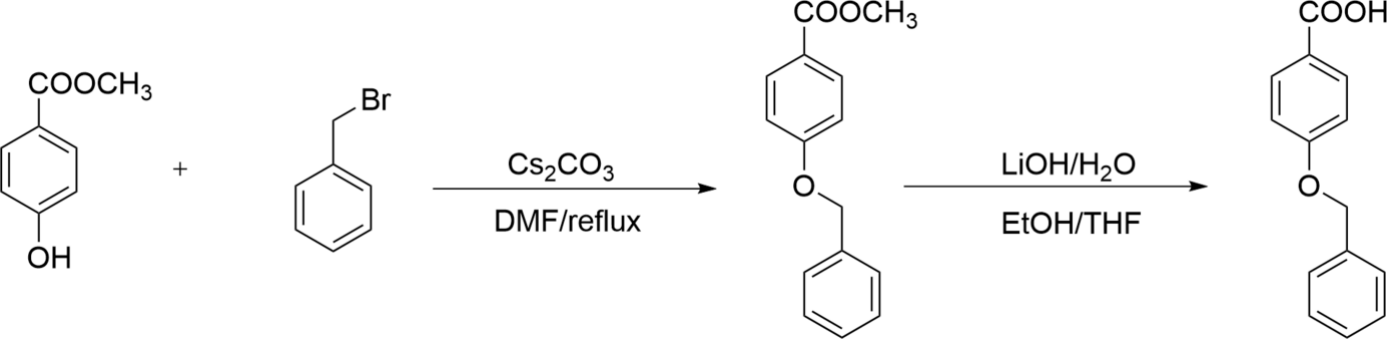
Synthesis of the title compound.

The title compound was prepared by a two-step reaction (Fig. 3 ▸). The product was purified by column chromatography. Single crystals were obtained by slowly evaporating an ethanol solution of the title compound.

## Refinement

Crystal data, data collection and structure refinement details are summarized in Table 2 ▸.

## Supplementary Material

Crystal structure: contains datablock(s) global, I. DOI: 10.1107/S2414314624007521/hb4473sup1.cif

Structure factors: contains datablock(s) I. DOI: 10.1107/S2414314624007521/hb4473Isup2.hkl

Supporting information file. DOI: 10.1107/S2414314624007521/hb4473Isup3.cml

CCDC reference: 2374704

Additional supporting information:  crystallographic information; 3D view; checkCIF report

## Figures and Tables

**Table 1:** Hydrogen-bond geometry (Å, °)

*D*—H⋯*A*	*D*—H	H⋯*A*	*D*⋯*A*	*D*—H⋯*A*
O1—H1⋯O2^i^	0.82	1.81	2.6213 (15)	169

Symmetry code: (i) 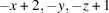.

**Table 2:** Experimental details

Crystal data	
Chemical formula	C_14_H_12_O_3_
*M*_r_	228.24
Crystal system, space group	Monoclinic, *P*2_1_/*n*
Temperature (K)	305
*a*, *b*, *c* (Å)	10.0564 (5), 3.9985 (2), 28.2235 (14)
β (°)	97.744 (5)
*V* (Å^3^)	1124.54 (10)
*Z*	4
Radiation type	Mo *K*α
μ (mm^−1^)	0.10
Crystal size (mm)	0.16 × 0.15 × 0.13

Data collection	
Diffractometer	XtaLAB Synergy R, DW system, HyPix
Absorption correction	Multi-scan (*CrysAlis PRO*; Rigaku OD, 2022 ▸)
*T*_min_, *T*_max_	0.875, 1.000
No. of measured, independent and observed [*I* > 2σ(*I*)] reflections	10523, 2793, 1996
*R*_int_	0.019
(sin θ/λ)_max_ (Å^−1^)	0.721

Refinement	
*R*[*F*^2^ > 2σ(*F*^2^)], *wR*(*F*^2^), *S*	0.044, 0.135, 1.08
No. of reflections	2793
No. of parameters	156
H-atom treatment	H-atom parameters constrained
Δρ_max_, Δρ_min_ (e Å^−3^)	0.26, −0.19

Computer programs: *CrysAlis PRO* (Rigaku OD, 2022 ▸), *SHELXT2018/3* (Sheldrick, 2015*b* ▸), *SHELXL2018/3* (Sheldrick, 2015*b* ▸) and *OLEX2* (Dolomanov *et al.*, 2009 ▸).

## full crystallographic data

### 4-(Benzyloxy)benzoic acid Crystal data

**Table:**

C_14_H_12_O_3_	*F*(000) = 480
*M**_r_* = 228.24	*D*_x_ = 1.348 Mg m^−^^3^
Monoclinic, *P*2_1_/*n*	Mo *K*α radiation, λ = 0.71073 Å
*a* = 10.0564 (5) Å	Cell parameters from 5298 reflections
*b* = 3.9985 (2) Å	θ = 2.1–29.8°
*c* = 28.2235 (14) Å	µ = 0.10 mm^−^^1^
β = 97.744 (5)°	*T* = 305 K
*V* = 1124.54 (10) Å^3^	Block, clear light colourless
*Z* = 4	0.16 × 0.15 × 0.13 mm

### 4-(Benzyloxy)benzoic acid Data collection

**Table:**

XtaLAB Synergy R, DW system, HyPix diffractometer	2793 independent reflections
Radiation source: Rotating-anode X-ray tube, Rigaku (Mo) X-ray Source	1996 reflections with *I* > 2σ(*I*)
Mirror monochromator	*R*_int_ = 0.019
Detector resolution: 10.0000 pixels mm^-1^	θ_max_ = 30.8°, θ_min_ = 2.1°
ω scans	*h* = −12→13
Absorption correction: multi-scan (CrysAlisPro; Rigaku OD, 2022)	*k* = −4→5
*T*_min_ = 0.875, *T*_max_ = 1.000	*l* = −29→37
10523 measured reflections	

### 4-(Benzyloxy)benzoic acid Refinement

**Table:**

Refinement on *F*^2^	Hydrogen site location: inferred from neighbouring sites
Least-squares matrix: full	H-atom parameters constrained
*R*[*F*^2^ > 2σ(*F*^2^)] = 0.044	*w* = 1/[σ^2^(*F*_o_^2^) + (0.0641*P*)^2^ + 0.1717*P*] where *P* = (*F*_o_^2^ + 2*F*_c_^2^)/3
*wR*(*F*^2^) = 0.135	(Δ/σ)_max_ < 0.001
*S* = 1.08	Δρ_max_ = 0.26 e Å^−^^3^
2793 reflections	Δρ_min_ = −0.19 e Å^−^^3^
156 parameters	Extinction correction: *SHELXL2018/3* (Sheldrick, 2015b), Fc^*^=kFc[1+0.001xFc^2^λ^3^/sin(2θ)]^-1/4^
0 restraints	Extinction coefficient: 0.008 (2)

### 4-(Benzyloxy)benzoic acid Special details

**Table:**

Geometry. All esds (except the esd in the dihedral angle between two l.s. planes) are estimated using the full covariance matrix. The cell esds are taken into account individually in the estimation of esds in distances, angles and torsion angles; correlations between esds in cell parameters are only used when they are defined by crystal symmetry. An approximate (isotropic) treatment of cell esds is used for estimating esds involving l.s. planes.
Refinement. The positions of the H atom attached to O1 was obtained from a difference Fourier map. Other H atoms were positioned geometrically with C—H = 0.93 Å and constrained to ride on their parent atoms with *U*_iso_(H) = 1.2*U*_eq_(C,*O*).

### 4-(Benzyloxy)benzoic acid Fractional atomic coordinates and isotropic or equivalent isotropic displacement parameters (Å^2^)

**Table:**

	*x*	*y*	*z*	*U*_iso_*/*U*_eq_	
O3	0.35692 (9)	0.6152 (3)	0.36054 (4)	0.0557 (3)	
O2	0.94382 (10)	0.2255 (4)	0.45018 (4)	0.0666 (4)	
O1	0.83138 (10)	0.0288 (4)	0.50696 (4)	0.0658 (4)	
H1	0.906153	−0.043118	0.517133	0.099*	
C4	0.46870 (13)	0.5035 (4)	0.38859 (5)	0.0444 (3)	
C5	0.58913 (14)	0.5572 (4)	0.37085 (5)	0.0503 (4)	
H5	0.589311	0.663292	0.341539	0.060*	
C6	0.70792 (13)	0.4545 (4)	0.39638 (5)	0.0491 (4)	
H6	0.788198	0.490876	0.384244	0.059*	
C1	0.70890 (12)	0.2960 (4)	0.44041 (4)	0.0431 (3)	
C2	0.58902 (13)	0.2464 (4)	0.45795 (5)	0.0495 (4)	
H2	0.589103	0.142972	0.487473	0.059*	
C3	0.46833 (14)	0.3477 (4)	0.43243 (5)	0.0505 (4)	
H3	0.388052	0.311700	0.444589	0.061*	
C8	0.22907 (13)	0.5294 (4)	0.37376 (5)	0.0526 (4)	
H8A	0.216021	0.642645	0.403176	0.063*	
H8B	0.224207	0.290205	0.378971	0.063*	
C9	0.12226 (13)	0.6340 (3)	0.33413 (5)	0.0452 (3)	
C10	0.13472 (15)	0.5661 (4)	0.28683 (5)	0.0548 (4)	
H10	0.211545	0.460996	0.279204	0.066*	
C11	0.03322 (16)	0.6542 (4)	0.25081 (6)	0.0616 (4)	
H11	0.042281	0.608856	0.219082	0.074*	
C12	−0.08060 (15)	0.8081 (4)	0.26183 (6)	0.0625 (4)	
H12	−0.148833	0.865897	0.237618	0.075*	
C13	−0.09369 (15)	0.8766 (4)	0.30845 (6)	0.0623 (4)	
H13	−0.170991	0.980303	0.315929	0.075*	
C14	0.00810 (14)	0.7917 (4)	0.34459 (6)	0.0536 (4)	
H14	−0.000845	0.841711	0.376181	0.064*	
C7	0.83567 (13)	0.1772 (4)	0.46735 (5)	0.0467 (3)	

### 4-(Benzyloxy)benzoic acid Atomic displacement parameters (Å^2^)

**Table:**

	*U*^11^	*U*^22^	*U*^33^	*U*^12^	*U*^13^	*U*^23^
O3	0.0376 (5)	0.0723 (7)	0.0548 (6)	−0.0030 (5)	−0.0018 (4)	0.0171 (5)
O2	0.0403 (5)	0.1045 (10)	0.0549 (6)	0.0051 (6)	0.0064 (4)	0.0152 (6)
O1	0.0449 (6)	0.0978 (9)	0.0527 (6)	0.0062 (6)	0.0000 (5)	0.0210 (6)
C4	0.0394 (7)	0.0492 (8)	0.0425 (7)	−0.0025 (6)	−0.0015 (5)	0.0019 (6)
C5	0.0461 (7)	0.0633 (9)	0.0407 (7)	−0.0034 (6)	0.0029 (6)	0.0094 (6)
C6	0.0378 (7)	0.0648 (9)	0.0447 (7)	−0.0040 (6)	0.0056 (5)	0.0046 (6)
C1	0.0390 (7)	0.0499 (8)	0.0392 (6)	−0.0014 (5)	0.0011 (5)	−0.0005 (5)
C2	0.0434 (7)	0.0629 (9)	0.0417 (7)	−0.0008 (6)	0.0038 (5)	0.0089 (6)
C3	0.0378 (7)	0.0644 (10)	0.0492 (8)	−0.0020 (6)	0.0058 (5)	0.0083 (7)
C8	0.0405 (7)	0.0618 (9)	0.0546 (8)	−0.0032 (6)	0.0025 (6)	0.0103 (7)
C9	0.0374 (6)	0.0455 (8)	0.0515 (8)	−0.0053 (5)	0.0019 (5)	0.0046 (6)
C10	0.0462 (8)	0.0608 (10)	0.0573 (8)	0.0017 (7)	0.0068 (6)	−0.0025 (7)
C11	0.0607 (9)	0.0722 (11)	0.0497 (8)	−0.0119 (8)	−0.0004 (7)	0.0001 (7)
C12	0.0449 (8)	0.0691 (11)	0.0689 (10)	−0.0100 (7)	−0.0094 (7)	0.0173 (8)
C13	0.0396 (7)	0.0666 (11)	0.0803 (11)	0.0039 (7)	0.0063 (7)	0.0117 (8)
C14	0.0438 (7)	0.0609 (9)	0.0568 (8)	−0.0024 (7)	0.0090 (6)	0.0022 (7)
C7	0.0406 (7)	0.0584 (9)	0.0403 (7)	−0.0004 (6)	0.0022 (5)	−0.0007 (6)

### 4-(Benzyloxy)benzoic acid Geometric parameters (Å, º)

**Table:**

O3—C4	1.3601 (16)	C3—H3	0.9300
O3—C8	1.4279 (17)	C8—H8A	0.9700
O2—C7	1.2636 (17)	C8—H8B	0.9700
O1—H1	0.8200	C8—C9	1.5019 (19)
O1—C7	1.2716 (17)	C9—C10	1.384 (2)
C4—C5	1.3881 (19)	C9—C14	1.376 (2)
C4—C3	1.3857 (19)	C10—H10	0.9300
C5—H5	0.9300	C10—C11	1.386 (2)
C5—C6	1.3721 (19)	C11—H11	0.9300
C6—H6	0.9300	C11—C12	1.372 (2)
C6—C1	1.3937 (19)	C12—H12	0.9300
C1—C2	1.3780 (18)	C12—C13	1.368 (2)
C1—C7	1.4723 (18)	C13—H13	0.9300
C2—H2	0.9300	C13—C14	1.386 (2)
C2—C3	1.3858 (19)	C14—H14	0.9300
			
C4—O3—C8	118.15 (11)	C9—C8—H8A	110.0
C7—O1—H1	109.5	C9—C8—H8B	110.0
O3—C4—C5	115.61 (12)	C10—C9—C8	121.00 (13)
O3—C4—C3	124.46 (12)	C14—C9—C8	120.09 (13)
C3—C4—C5	119.93 (12)	C14—C9—C10	118.89 (13)
C4—C5—H5	119.9	C9—C10—H10	119.9
C6—C5—C4	120.30 (12)	C9—C10—C11	120.29 (14)
C6—C5—H5	119.9	C11—C10—H10	119.9
C5—C6—H6	119.8	C10—C11—H11	119.9
C5—C6—C1	120.36 (12)	C12—C11—C10	120.15 (15)
C1—C6—H6	119.8	C12—C11—H11	119.9
C6—C1—C7	120.58 (12)	C11—C12—H12	120.0
C2—C1—C6	118.97 (12)	C13—C12—C11	119.97 (14)
C2—C1—C7	120.44 (12)	C13—C12—H12	120.0
C1—C2—H2	119.4	C12—C13—H13	120.0
C1—C2—C3	121.23 (13)	C12—C13—C14	120.10 (15)
C3—C2—H2	119.4	C14—C13—H13	120.0
C4—C3—C2	119.21 (13)	C9—C14—C13	120.60 (14)
C4—C3—H3	120.4	C9—C14—H14	119.7
C2—C3—H3	120.4	C13—C14—H14	119.7
O3—C8—H8A	110.0	O2—C7—O1	122.78 (12)
O3—C8—H8B	110.0	O2—C7—C1	118.87 (12)
O3—C8—C9	108.51 (11)	O1—C7—C1	118.36 (12)
H8A—C8—H8B	108.4		
			
O3—C4—C5—C6	179.92 (14)	C2—C1—C7—O1	0.0 (2)
O3—C4—C3—C2	−179.61 (14)	C3—C4—C5—C6	0.5 (2)
O3—C8—C9—C10	45.48 (19)	C8—O3—C4—C5	172.07 (13)
O3—C8—C9—C14	−136.32 (14)	C8—O3—C4—C3	−8.5 (2)
C4—O3—C8—C9	−171.59 (12)	C8—C9—C10—C11	177.81 (14)
C4—C5—C6—C1	−0.2 (2)	C8—C9—C14—C13	−177.25 (14)
C5—C4—C3—C2	−0.2 (2)	C9—C10—C11—C12	−0.3 (3)
C5—C6—C1—C2	−0.4 (2)	C10—C9—C14—C13	1.0 (2)
C5—C6—C1—C7	178.37 (14)	C10—C11—C12—C13	0.4 (3)
C6—C1—C2—C3	0.7 (2)	C11—C12—C13—C14	0.2 (3)
C6—C1—C7—O2	0.9 (2)	C12—C13—C14—C9	−0.9 (2)
C6—C1—C7—O1	−178.79 (15)	C14—C9—C10—C11	−0.4 (2)
C1—C2—C3—C4	−0.4 (2)	C7—C1—C2—C3	−178.11 (14)
C2—C1—C7—O2	179.73 (14)		

### 4-(Benzyloxy)benzoic acid Hydrogen-bond geometry (Å, º)

**Table:**

*D*—H···*A*	*D*—H	H···*A*	*D*···*A*	*D*—H···*A*
O1—H1···O2^i^	0.82	1.81	2.6213 (15)	169

Symmetry code: (i) −*x*+2, −*y*, −*z*+1.
